# Strategic considerations on developing a CHIKV vaccine and ensuring equitable access for countries in need

**DOI:** 10.1038/s41541-023-00722-x

**Published:** 2023-08-18

**Authors:** Neil Cherian, Alison Bettis, Arminder Deol, Arun Kumar, Jose Luis Di Fabio, Amol Chaudhari, Solomon Yimer, Raafat Fahim, Timothy Endy

**Affiliations:** Coalition for Epidemics Preparedness Innovations, Oslo, Norway

**Keywords:** Infectious diseases, Drug development

## Abstract

Chikungunya is an arboviral disease caused by the chikungunya virus (CHIKV) afflicting tropical and sub-tropical countries worldwide. It has been identified as a priority pathogen by the Coalition for Epidemics Preparedness Innovations (CEPI) and as an emerging infectious disease (EID) necessitating further action as soon as possible by the World Health Organization (WHO). Recent studies suggest that disability-adjusted life years (DALYs) due to CHIKV infection are as high as 106,089 DALYs lost globally. Significant progress has been made in the development of several vaccines, aimed at preventing CHIKV infections. This perspective article summarizes CEPI’s efforts and strategic considerations for developing a CHIKV vaccine and ensuring equitable access for CHIKV endemic countries.

## Introduction

Chikungunya is an infectious disease with a significant burden especially affecting the tropical Global South. There are at present no licensed vaccines, although several advanced candidates are approaching the approval process. The lack of long-term surveillance programs and corresponding data in chikungunya-affected/endemic regions such as Africa, Latin America, and Southeast Asia as well as the sporadic transmission and unpredictable outbreak patterns of CHIKV have impeded conduct of randomized controlled efficacy trials. This impediment, alongside the established disease morbidity and burden, have led authorities to explore alternative data provisions to facilitate development and licensure of medical products/vaccines for CHIKV. Mature regulatory authorities, including the Food and Drug Administration (FDA) and European Medicines Agency (EMA), recognize the substantial unmet medical need posed by Chikungunya disease and the limited feasibility of Phase 3 efficacy trials and are supportive of alternative licensure pathways (e.g., Accelerated approval/PRIME), using a surrogate marker of protection^1,2^.

Here, we introduce the Coalition of Epidemic Preparedness Innovations (CEPI’s) funding considerations to facilitate access, supply, and outbreak response of licensed vaccines and summarize our approach to support registration of CHIKV vaccines in endemic countries. We also highlight the need for multilateral and normative institutions to begin preparing mechanisms for the acceptance, procurement, and uptake of licensed CHIKV vaccines, beyond the limited scope and mandate of CEPI funding. Given the epidemic and outbreak potential of CHIKV we also highlight applications of lessons learned to future outbreak response building on regulatory and manufacturing innovations and regional use scenarios.

## Current overview of vaccine candidate pipeline in Phase 2&3 and CEPI CHIKV investments

Multiple CHIKV vaccines are in development from the pre-clinical stage to Phase 1, 2, and 3^3,4^. CEPI is funding the progress of Phase 2–3 candidates into advanced clinical development and eventual licensure of vaccines for use in affected regions. With support from the European Commission (EC), CEPI has invested in candidates showing promising results in Phase 1 and 2 clinical trials (Table 1). Amongst vaccines in the pipeline, a live attenuated CHIKV vaccine developed by Valneva (with CEPI support) has completed Phase 3 studies and is nearing licensure in the US and EU followed by other endemic regions. An inactivated two-dose vaccine developed by Bharat Biotech International Limited, also with CEPI support, is in Phase 2/3 clinical trials with the goal of licensure in India. Emergent Biosciences, while not in the CEPI portfolio, has a virus-like particle (VLP) vaccine candidate in a Phase 3 trial and recently received a US Department of Defense (DoD) grant for a post-licensure trial in an endemic region (Thailand), indicating that licensure is likely in the near term^5^. In 2023, this VLP candidate was purchased by Bavarian Nordic, the manufacturer of the Jynneos MPox vaccine^6^. CEPI also supported the Themis (MSD) Measles-Vectored CHIK program which was put on hold after completion of a Phase 2 study^7^.

**Table 1:** Leading CHIKV vaccine candidates and Phase 2–3 clinical approaches to licensure.

Candidate name and Developer	Technology approach and viral strain	Dosing regimen	Clinical stage	Clinical trial overview	Clinical trial references	NHP challenge study	Anticipated licensure pathway	Regulatory incentives for development	Post licensure needs
VLA1553, Valneva VLA1555, Instituto Butantan	Recombinant LA CHIKV with nsP3 deletion (La LR2006 OPY1, Δ5nsP3)	Single dose	Phase 3	• Phase 3 pivotal (US) – completed • Phase 3 Lot-to-Lot study – completed • Phase 3b^1^ (Brazil, 12–15ys) – ongoing	NCT04546724 NCT04650399 NCT04786444	Roques et al.^10^ CHIKV RNA μPRNT50 titer of ≥ 150	• FDA Accelerated approval pathway^3^ • EMA MAA using clinical safety and immunogenicity data supported by NHP surrogate indicator of protection • ANVISA licensure	• FDA Breakthrough therapy designation • FDA Fast Track designation • EMA Priority Medicine (PRIME) designation	• Phase 4 effectiveness study • Phase 3b study for immunocompromised and pregnant subgroups • Phase 3b for pediatric populations
CHIKV-VLP/ PXVX0317, Emergent BioSolutions	Virus-like-particles with alhydrogel adjuvant (CHIKV strain 37997, West African lineage)	Single dose	Phase 3	• Phase 3 pivotal (US) – ongoing • Phase 3b (US, Thailand, > 65 yrs) - ongoing	NCT05072080 NCT05349617	Akahata et al.^43^ CHIKV PRNT50 titers ≥ 20 considered positive	• FDA Accelerated approval pathway • EMA MAA using Ph3 Safety and immunogenicity with Serum Neutralizing Antibodies	• FDA Breakthrough therapy designation • FDA Fast Track designation • EMA Priority Medicine (PRIME) designation	• Post-Approval Field Efficacy Study^4^
BBV87, Bharat Biotech	Inactivated whole virion CHIKV (CHIK 03/06 India, ECSA lineage)	2-dose at 0 and 28 days	Phase 2b	• Phase 2/3 (India) – ongoing • Phase 2/3^1^ (Thailand, Panama, Colombia, Guatemalsa, Costa Rica) – ongoing	CTRI/2021/09/036179 NCT04566484	Studies completed^2,1^: • NHPs protected from viremia after Passive immunization with Ph2 vaccinee sera and CHIKV challenge • NHPs protected from viremia after active immunization with BBV87 and CHIKV challenge	• DCGI licensure using Ph3 safety and immunogenicity supported by Active and Passive immunization in NHPs	N/A	N/A
MV-CHIK-202/V-184 Themis biosciences -MSD	Measles Vectored CHIKV structural proteins (pTMMVSchwCE3E26KE1)	2-dose at 0 and 28 days	Phase 2	• Phase 2^1^ (Austria, Germany) – completed • Phase 2a^1^ (Puerto Rico, US) - completed	NCT02861586 NCT03101111	Rossi et al.^44^ CHIKV RNA PRNT50 titer of ≥ 20	• FDA Accelerated approval pathway • EMA	• FDA Breakthrough therapy designation • FDA Fast Track designation • EMA Priority Medicine (PRIME) designation	Program put on hold in Feb 2023
								First Developer to successfully submit a BLA to US FDA qualifies for a Priority Review Voucher (PRV), representing a significant financial incentive which is transferable and holds significant value	

^1^CEPI-EC funded.

^2^Pending publication.

^3^Submission completed with clinical safety and immunogenicity data supported by NHP surrogate indicator of protection, in lieu of Phase 3 efficacy data.

^4^US DoD funded.

**Fig. 1:**
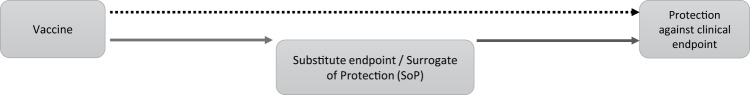
Relationship between vaccine, substitute endpoint (SoP) and clinical endpoint. Adapted from Correlates of vaccine-induced protection: methods and implications, WHO IVR-IVB, 2013.

Notably, FDA/Center for Biologics Evaluation and Research (CBER) has endorsed an accelerated approval process for Valneva’s live attenuated CHIKV vaccine (VLA1553)^8^, which uses a surrogate endpoint of protection. The FDA defines a surrogate endpoint as a “marker, such as a laboratory measurement, radiographic image, physical sign or other measure that is thought to predict clinical benefit but is not itself a measure of clinical benefit” (Fig. 1). This position was reflected and discussed in FDA’s Vaccines and Related Biological Products Advisory Committee (VRBPAC) summary from November 2019^9^, and has played a key role in enabling a licensure pathway for advanced CHIKV vaccines. For VLA1553, the surrogate was established in a non-human primate (NHP) infection model to establish a level of biomarker induced by a vaccine candidate that is demonstrative of protection from viral infection and/or challenge^10^. Non-human primate CHIKV infection models have emerged as valuable tools for investigating CHIKV infection, assessing vaccines in preclinical studies, and establishing an immune correlate of protection to support regulatory needs for licensure. Data from a sero-epidemiological study performed in the Philippines^11^ demonstrated that participants with even a very low-positive neutralization titer (PRNT80 ≥ 10) were protected from symptomatic CHIKV infection. Developers have extrapolated this data point to their respective assays and derive a surrogate threshold of protection using challenge studies in an NHP model of infection. This approach has been complemented by other incentives such as breakthrough therapy designation, fast track designation, and financial incentives such as priority review vouchers to incentivize product/vaccine developers to fulfill an unmet need in a neglected disease area^12^.

Similarly, EMA has endorsed three CHIKV vaccine candidates (i.e., Valneva, Themis [MSD], PaxVax [Emergent-BN]) candidates for its PRIME scheme^13^. The latter enhances support for priority medicines that target an unmet medical need supported by a surrogate (non-clinical) marker of protection.

## Further R&D work required for completion of clinical development of most advanced candidates

As developers proceed with clinical testing in Phase 3 (and likely Phase 4 trials post-licensure), ensuring the quality, standardization, and harmonization of laboratory testing of clinical samples is crucial for accurate assessment of vaccine efficacy. A diverse array of assays are employed to measure neutralizing antibodies, incorporating distinct viruses or antigens and variable operational and reporting methods. Thus, comparison of results is difficult. The World Health Organization (WHO) has recently endorsed an international antibody standard to harmonize immune response ranges across multiple vaccine trials. The WHO-endorsed standard, developed by the Paul Ehrlich Institute (PEI) in 2022 using an East/Central/South African strain of CHIKV, can facilitate the use of a uniform biomarker for immune response measurement and is readily available for use^14^.

Incorporation of a harmonized standard would ensure accuracy and consistency across different vaccines, and also distinguish between immune response characteristics for each vaccine. Product characteristics can inform vaccine use in varying scenarios such as outbreak versus routine use, geographical suitability in terms of costs, and future response scenario planning and procurement planning for Ministries of Health and multilateral agencies such as PAHO (Pan American Health Organization), UNICEF (United Nations Children’s Fund), and GAVI (Global Alliance for Vaccines and Immunization) amongst others.

Incorporation of the established international standard into the testing will be crucial to measure vaccine performance and provide additional data for suitability to specific groups (i.e., pediatric, immunocompromised, pregnant populations) if multiple candidates are assessed.

## Epidemiological gaps and modeling needs to facilitate Phase 3 efficacy trials

Knowledge gaps in CHIKV epidemiology hamper the preparation, site selection, and implementation of CHIKV vaccine efficacy trials. CHIKV has a wide geographic distribution, and available information tends to focus on large-scale outbreaks with historically few examples describing low-level or endemic transmission. As outbreaks are generally not fully characterized, details of age-specific incidence are often limited. With the example of the recent outbreak in Paraguay, the unusual pattern of chikungunya virus outbreaks and the prevalence of immunity in affected populations highlight the challenges of evaluating candidate vaccines in a Phase 3 trial.

Many CHIKV endemic areas are co-endemic for arboviruses sharing similar clinical presentations (e.g., Dengue, Zika). A lack of specific case definitions to differentiate between these arboviruses, as well as a low proportion of lab-confirmed cases during outbreaks (especially in resource-poor settings) means that co-infection and/or misclassification is likely and could contribute to under- or over-reporting of chikungunya cases.

While there are gaps in understanding of long-term population immunity and duration of population protection, evidence of durable protection following infection with Ross River virus (RRV), a related virus^15^, could be applied to CHIKV. Available data (particularly seroprevalence studies) suggest that CHIKV outbreaks are unlikely to occur in areas which have recently experienced a large-scale outbreak^16^ but provide insufficient information to characterize the duration of population protection.

Many CHIKV-endemic countries do not have continuous CHIKV-focused surveillance systems in place, as many systems are active for a limited time and/or in a limited geographic area^16^. While some insight can be gleaned from surveillance of other arboviruses (e.g., dengue), the fact that most surveillance activities are clinic- or hospital-based and rely primarily on clinical diagnosis (rather than laboratory confirmation) means that granularity of transmission dynamics between arboviruses with similar clinical presentation (DENV, ZIKV, CHIKV) is lacking. Available ongoing surveillance reports, such as chikungunya situation reports from the Government of India’s National Center for Vector-Borne Diseases Control^17^, Brazil’s weekly reporting^18^, and the weekly chikungunya reports released by PLISA Health Information Platform of the Americas^19^ often share limited information publicly (not including surveillance methods, case definitions, diagnostic tests used to confirm cases). Working directly with endemic countries and key stakeholders (e.g., PAHO) could provide valuable depth to publicly available data. Improved and sustained surveillance of CHIKV in endemic areas, including laboratory confirmation of cases, would greatly improve our understanding of CHIKV disease dynamics and enable the planning and implementation of CHIKV vaccine efficacy trials. While some recent examples describing inter-epidemic or endemic transmission of CHIKV in Kenya^20^ and Thailand^21^, further characterization of endemic transmission in key areas could provide valuable insight, as there is insufficient evidence to suggest that vaccine effectiveness trials would be feasible in such areas.

Mathematical modeling utilizing a range of data sources (e.g., seroprevalence data, case data, mosquito data) could also contribute valuable insight and more granular estimates of disease burden. The disease distribution information can be used to create CHIKV risk maps to aid in planning for vaccine efficacy trials. Additionally, models can be used to predict possible future disease burden and outbreaks, inform vaccine demand, predict vaccine impact, and determine optimal vaccination strategies (and test others) under different epidemiological scenarios^22^.

Given the recent progress in CHIKV vaccine development, the knowledge gaps described above may not be addressed ahead of the licensure of a CHIKV vaccine. Filling these epidemiological knowledge gaps remains important for post-licensure activities such as Phase 4 effectiveness studies, which will inform governments’ go/no-go decision on national inclusion of any CHIKV vaccine and in turn inform demand for and equitable access to a CHIKV vaccine.

## Currently ongoing Phase 3 clinical trial designs and expected endpoints, alternative approaches to licensing and post-licensure requirements

The WHO suggests a Phase 3 prospective, double-blind, placebo-controlled, efficacy trial for the evaluation of a chikungunya vaccine based on the primary endpoint of laboratory confirmed chikungunya illness. The planning and conduct of such a large trial, however, is challenging due to aforementioned reasons and this has been acknowledged by WHO^23^. Under these circumstances WHO considers the possibility of deriving an immunological marker of protection from non-clinical efficacy trials or passive protection trials – that is, nonclinical or clinical trials which assess the effects of administering normal or hyperimmune human gamma globulin or convalescent sera and the establishment of a minimum protective antibody level sufficient for the prevention of clinical disease that can be used to interpret data obtained in clinical trials with the candidate vaccines. This is reflected in the choice of study design of pivotal Phase 3 clinical trials of advanced vaccine candidates (Table 1).

Valneva’s pivotal, placebo-controlled, Phase 3 clinical trial of its live attenuated vaccine candidate (VLA1553) evaluated the safety and immunogenicity following a single dose among adults (*n* = 4115). The primary outcome measure was the rate of seroprotection at 28 days following vaccination in baseline seronegative participants. Seroprotection was defined as anti-CHIKV antibody titers based on a µPRNT assay that were above the level of ‘surrogate of protection’ agreed with US FDA. Results of this study formed the basis of Biologics License Application (BLA) to the US FDA for approval of the vaccine for use in adults aged 18 years and above^24^. In addition, Valneva, in collaboration with Instituto Butantan is conducting a Phase 3 clinical trial among adolescents—data from which may be used for Brazilian regulatory approval in this population. The trial’s primary objective is based on immunogenicity at 28 days following immunization.

Bharat Biotech’s inactivated vaccine candidate (BBV87) is undergoing a pivotal Phase 2/3 clinical trial in multiple countries in Latin America (Panama, Colombia, Costa Rica, Guatemala) and Asia (Thailand) among 12–65-year-olds (*n* = 3210). Using a placebo-controlled, randomized, adaptive, seamless study design, allows for modification based on dose selection done in early parts of the trial followed by a dose confirmation in later phases. The primary outcome measure is geometric mean titers (GMTs) of neutralizing antibodies at 28 days following a second vaccine dose. This trial is sponsored by the International Vaccine Institute. A parallel Phase 2/3 trial with similar primary outcome measures and study population is underway in India, sponsored by Bharat Biotech.

Emergent BioSolutions’ VLP-based vaccine candidate (CHIK VLP) is being evaluated in a placebo-controlled Phase 3 clinical trial in USA among 12–65-year-old healthy seronegative individuals (*n* = 3258). It will also demonstrate the immunogenic consistency of three different manufacturing lots. The primary outcome measure is based on GMTs of neutralizing antibodies against CHIKV at 21 days following vaccination. A parallel Phase 3 trial is underway to assess immunogenicity in adults > 65years (*n* = 413).

Other vaccine candidates are yet to move to the pivotal (Phase 3) stage of clinical development. All three aforementioned developers are conducting pivotal clinical trials—that form the basis of approval for use in general population—utilizing immunogenicity endpoints. Emergent has planned for a post-licensure trial^5^, but public information on other developers’ plans is limited.

With multiple genotypes of CHIKV in circulation such as East/Central/South African, West African, and Asian strains, cross-neutralization across lineages has been demonstrated^9,25^, and will likely need to be established with each individual vaccine candidate for regulatory authorization.

Considering that novel chikungunya vaccines may be authorized soon, plans for measuring effectiveness post-approval need discussion with regulators to gain good understanding of data to be generated after licensure. In addition, developers with CHIKV vaccine programs nearing licensure should plan human clinical trials in special subgroup populations to include age de-escalation into pediatric populations, pregnant women, and immunocompromised subjects, further expanding utility of licensed vaccines including in CHIKV outbreaks.

## Diagnostic needs—rapid diagnostics tests (RDTs) and validated assays for Ph4 effectiveness trials

The design of effectiveness studies for CHIKV vaccine may vary depending on the objectives and endpoints set by respective vaccine developers. As a result, the diagnostic requirements will also vary depending on effectiveness study design. The tests needed may include highly sensitive, specific, and validated RT-PCR assay for case confirmation, and immunoassays (IgG, IgM ELISA) for assessing vaccine induced immunity or prior exposure to a natural infection. Rapid diagnostics tests (RDTs) will be useful for early detection of an outbreak. Specifically, high performing and validated molecular RDTs will be extremely important for participant recruitment in rural areas where RT-PCR test is often unavailable.

Landscape analyses of CHIKV diagnostics have identified commercially available conventional and real-time (RT-PCR) test, isothermal and multiplex PCR assays^26,27^. However, the performance of the available molecular tests has not been clinically validated. No RT-PCR test has received full regulatory approval (FDA) for CHIKV detection to date. With multiple chikungunya virus lineages circulating globally^28^, confirmation that available RT-PCR tests are cross-sensitive and specific across lineages/clades is needed. The performances of available RT-PCR tests for CHIKV also needs clinical validation against other closely related arboviruses including DENV, ZIKV, Yellow fever virus (YFV), and West Nile virus (WNV), and alphaviruses (Mayaro virus [MAYV] and O’Nyong Nyong virus [ONNV]). With regards to immunoassays, there are several in-house and commercially available ELISA tests for the detection of CHIKV infection^26,27^, however, the performance of these assays have not passed through an independent clinical validation. In addition, significant cross-reactivity between anti-CHIKV and anti-ONNV antibodies, as well as between anti-CHIKV and anti-MAYV has been documented. To date, there is no reliable serology assay that can distinguish between these viruses in CHIKV endemic areas; and are needed to enable Phase 4 effectiveness studies.

Among other diagnostic tests useful for chikungunya effectiveness studies are RDTs, for use in participant screening and recruitment for Phase 4 effectiveness studies. In addition, RDTs have a significant contribution in early detection of cases and outbreaks, especially in rural areas with limited infrastructure to properly diagnose and manage patients. Peripheral health facilities (low level) without advanced laboratory equipment and skilled personnel to perform RT-PCR tests would benefit from the availability of a high-performing and validated RDT to allow early detection and management of chikungunya cases. It will also contribute to the recruitment of Phase 4 chikungunya vaccine effectiveness study participants from rural areas where RT-PCR is not available. A recent landscaping on chikungunya RDTs showed the commercially available antibody and antigen based RDTs^29^, but their performance is claimed to be variable. While antigen based RDTs are in development, there are no nucleic acid based RDTs for CHIKV to date.

For clinical confirmation of chikungunya, it is preferable to use RT-PCR during the first week of illness. However, as viremia declines after the first week of symptoms, an anti-chikungunya virus IgM test is the best alternative for diagnosis. IgM is detectable between 2–3 months after infection^30^. In addition, the presence of polyarthralgia may be considered as an important clinical diagnostic marker since most chikungunya cases experience arthralgia ranging from weeks to months. Specifically, it is important to combine an anti-chikungunya IgM test and clinical symptoms (polyarthralgia) for accurate diagnosis.

A highly sensitive, specific, and validated RDT could be used for Phase 4 effectiveness studies. Specifically, the development and validation of a high performing, automated, self-contained chikungunya nucleic acid-based test for use at peripheral health facilities is of high importance. This work could potentially be addressed by FIND, the diagnostic alliance.

## Regulatory considerations for National Regulatory Authorities and the road to WHO PQ

WHO has two procedures to determine the acceptability of vaccines and other products based on evaluation of quality, safety, efficacy, and performance, namely, the WHO Emergency Use Listing (EUL) and WHO Prequalification (PQ)^31^. Listing of products evaluated by these mechanisms assist UN agencies and Member States in procurement of these essential goods. The WHO EUL facilitates the availability of products for serious or immediately life threatening for pathogens with potential of causing an outbreak, epidemic or pandemic, while WHO PQ ensures that vaccines used in immunization programs are safe and effective.

Vaccines deemed eligible for WHO PQ are listed in the Vaccines PQ Priority List after discussions with UNICEF, the PAHO Revolving Fund, and other international procurement agencies and is based on the demand of UN supplied markets (national immunization programs), suitability for WHO programmatic needs, supply security and recommendations from WHO’s Strategic Advisory Group of Experts on Immunization (SAGE). The current list does not include CHIKV vaccines. It is reviewed every two years and inclusion in following updates are subject to SAGE review, recommendation, and a Director General’s endorsement. To date, CHIKV disease and vaccines are not included in SAGE’s agenda for discussion and recommendations thus not setting the stage for establishing PQ eligibility and consequent procurement of these vaccines by multilateral procurement bodies such UNICEF, GAVI and the PAHO Revolving fund for supply and use in developing/affected countries – all of which require WHO PQ as a criterion for purchase of medicinal products.

Thus, endemic countries or those where manufacturing developments are ongoing may need to anticipate appropriate regulatory pathways for vaccine licensure until a WHO PQ pathway for CHIKV vaccines is available. It is now a priority to present CHIKV vaccines as a control or prevention tool for CHIKV disease to organizations such as GAVI, UNICEF, PAHO and the Revolving fund.

Whilst guidelines on production, quality, safety, and efficacy evaluation from EMA and US FDA for regulation of some CHIKV vaccines serve as an example^32^, limited similar advice from WHO & SAGE are needed to establish guidelines for the regulatory processes by national regulatory authorities from LMICs.

In the meantime, promoting the understanding of the difficulties for the evaluation of CHIKV vaccines efficacy with National Regulatory Authorities and the acceptance of using surrogate markers of protection (SoP) can accelerate the availability of chikungunya vaccines in endemic countries and/or have them ready for use in case of outbreaks. It is encouraging that USFDA and EMA have accepted developers’ approach to establish a protective neutralizing antibody threshold through passive transfer in NHP’s with data from sero-epidemiological studies despite the limitations of a SoP. These limitations largely center on an appropriate animal model, in the case of CHIKV a non-human primate model, and its ability to translate to human protection. To date, only one study has demonstrated protective CHIKV neutralizing antibody levels in a prospective cohort study^33^. Despite these limitations, both regulatory agencies indicate that the establishment of the SoP is specific to each vaccine and populations in which it has been established.

In December 2021, PAHO, ANVISA, and CEPI convened a meeting of the NRAs (National Regulatory Agencies) of reference for PAHO to discuss the developments of chikungunya vaccines and share the regulatory experiences of the USFDA and EMA in their approaches to licensing these vaccines. Participant regulators were in agreement on the concept of approval based on SoP but indicated that they would require review of current regulations. They reiterated the importance of plans for completion of post-marketing effectiveness and safety data, and highlighted the need for more sero-epidemiological studies to support NHP studies and for a better basis for post-licensure effectiveness studies^34^.

Regulators also agreed to undertake joint and collaborative reviews using new or already existing mechanisms. This approach in the Americas could be reproduced in other regions to support chikungunya vaccines becoming available in endemic countries and generate useful information on safety and efficacy/effectiveness that will complement inclusion on the WHO PQ priority list of vaccines.

## Procurement of licensed vaccines before and after WHO PQ

WHO prequalification (PQ) is a critical route used to provide qualified, safe, and efficacious vaccines for United Nations’ (UN) procurement agencies and to countries for bulk purchasing and distributing medicines in resource-limited settings. The process for establishing eligibility for WHO PQ for procurement is therefore a crucial mechanism to facilitate development and access to medical products worldwide, and especially to countries with nascent regulatory capacity^35^.

**Fig. 2:**
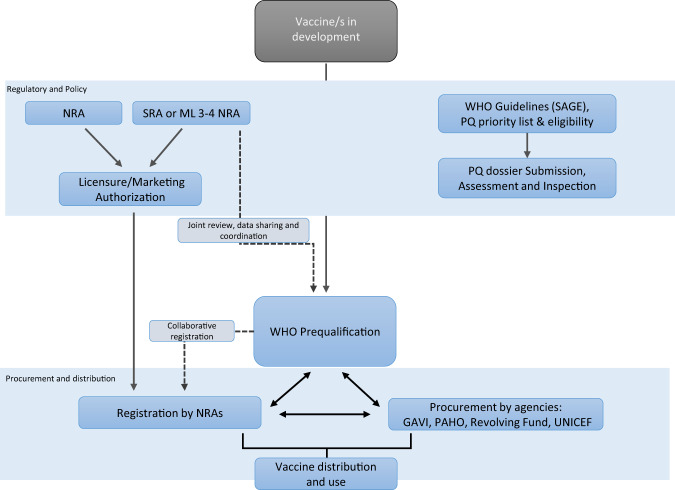
Overview of WHO PQ process for Vaccines. Adapted from Navigating Complexity to Improve Global Access, 2022.

The PQ prioritization process requires multiple rounds of consultation, advocacy, and final approval by the WHO Director General’s office to enable WHO prequalification eligibility of CHIKV vaccines for procurement and supply, especially in developing regions. Figure 2 presents an overview of this process and steps involved^36^. This status quo leaves countries to rely on approvals by external NRAs for local procurement and supply, until a vaccine is licensed locally or pre-qualified by WHO—significantly delaying access.

CHIKV vaccine candidates are uniquely positioned to address unmet medical need, whilst simultaneously constrained by lack of an established route to pre-qualification and licensure in LMICs. Given the financial incentives set in place by NRAs such as Accelerated Approval, Breakthrough designations (FDA), PRIME (EMA), etc., this leaves the onus of facilitating access in CHIKV endemic regions on the developer and is a gap requiring address. Given the large volume procurement that PQ facilitates, the WHO PQ pathway represents a significant financial incentive for developers and can be leveraged to expand access.

Furthermore, the low case fatality rate of chikungunya infections has also influenced the prioritization of the disease, with CHIKV missing a place on the influential WHO R&D Blueprint priority pathogen list despite having a significant and well document disease burden in multiple regions. Both, WHO R&D Blueprint Prioritization process and the PQ prioritization procedures are independent processes with varying advisory bodies and accountability structures within WHO.

WHO has recently launched its Global Arboviral initiative to address gaps in arthropod borne viral disease including those with a larger disease burden including DENV (Dengue virus), YFV (Yellow fever virus), ZIKV (Zika virus) and CHIKV, which brings together key partners and setting the stage for a broad strategy to address gaps in surveillance, preparedness and response and envisions engaging a wide variety of stakeholders to address identified gaps^37^. WHO has also commissioned a vaccine value profile that will likely be published in 2023^38^. More recently WHO has included CHIK as a neglected tropical disease that will be addressed as part of its 2030 disease strategy^39^.

In the near term, this status quo leaves the world exposed to an obvious vaccine inequity dilemma where only travelers and people in HIC (High Income Country) countries are able to access licensed vaccines. There is a clear need for interim measures to facilitate regional licensure in affected countries prior to availability of a PQ pathway for CHIKV vaccines. One example would be the EMA’s M4all Procedure (previously Article 58), which combines EMA’s scientific review capabilities with WHO expertise on local epidemiology and disease alongside involvement of national regulators in target countries. The process facilitates prequalification by WHO and can also support licensure in countries where expertise is nascent^40^.

## Key issues on access and supply of licensed vaccines to endemic regions

Vaccine developers with CHIKV candidates in Phase 3 studies, near national regulatory licensure or licensed by a national regulatory agency will likely need to execute an extended (5 years or more) Phase 3 human clinical trial and/or Phase 4 studies to facilitate access to products. Phase 3/4 studies will be crucial to establish long-term safety of the vaccine, durability of a surrogate marker of protection based on neutralizing antibody titers, and vaccine effectiveness through surveillance for virologically confirmed disease (using RT-PCR). Ideally, these studies will be performed in CHIKV endemic countries and linked to country-specific NRA requirements for approval and use in the local population.

Given limited guidance on CHIKV vaccines, there is minimal understanding of how endemic countries aim to use CHIKV vaccines within their local jurisdictions, i.e., whether incorporation into routine EPI (Expanded Programme on Immunization) programs or as an outbreak response vaccine, volumes of vaccines needed for either use scenario, and the willingness-to-pay and procurement interest. These are key gaps that need to be explored and addressed as developers bring their products to market. CEPI recently commissioned a Request for Proposals to identify these needs and is in the process of gathering this information^41^. Complementarily, key stakeholders such as WHO, GAVI, UNICEF will need to inform and prepare pathways to successfully facilitate acceptance, procurement, and uptake of licensed CHIKV vaccines.

To support transition to licensure and commercial supply, CEPI and regional authorities have begun engaging on local needs and acceptability of regulatory pathways for CHIKV vaccines and needs for post licensure data such as effectiveness data^34^. In addition, CEPI also convened a Joint Coordination Group in February 2022 to engage key stakeholders on facilitating access to CHIK vaccines, such as WHO, UNICEF, GAVI, AVAREF (Africa Vaccine Regulatory Forum), and FDA among others^42^.

Developers’ strategies to facilitate access to their respective products, could potentially incorporate technology transfer for manufacturing to endemic partners. If technically and commercially feasible, availability of vaccines in a local regulatory ecosystem would enable faster and more quality controlled supply of vaccines in an endemic setting. This approach has been adopted by Valneva through a technology transfer to Instituto Butantan in order to manufacture and supply vaccines to Brazil and the surrounding regions affected by CHIKV. Such technology transfers will require supportive evidence generation in endemic populations, immunocompromised and pediatric populations while expanding the overall product indication.

## Applications for the next Disease-X, and potential needs for outbreak response

As countries begin to prepare and develop programs targeting the next pandemic, a number of lessons from CHIKV vaccine development can be applied to preparing for the next Disease-X response. The vector-based transmission model and CHIKV’s ability to adapt to new mosquito vectors alongside emerging climate change patterns and migration of mosquito populations highlight the potential of a next large-scale epidemic or pandemic if conditions allow.

Lessons learned from CHIKV for the next Disease-X outbreak include its explosive nature of outbreaks with subsequent rapid decline of cases and the impact this has on trying to establish the clinical efficacy of future vaccines through formal RCTs. In the CHIKV case, the alternative pathway was to develop and promote the regulatory acceptance of a surrogate marker of protection based on animal studies as reasonably likely to predict clinical efficacy, followed by post-licensure commitments to provide safety and effectiveness data at the time of the next outbreak. Although these pathways are available through the US FDA, EMA, and other mature regulatory authorities, they must also be advocated as part of emergency preparedness plans of NRAs from LMICs. Regional regulatory engagements to reach consensus on emergency use and/or licensure of a vaccine for LMICs should be encouraged. Investments in the technology transfer of LMIC manufacturing and vaccine distribution, supported by enhancement of surveillance programs and diagnostics for regional disease threats will also be critical to a successful response.

Lastly, the ability to utilize a SoP as a measurement of efficacy for CHIKV vaccines can be applied to develop and deploy a vaccine for the next Disease-X outbreak. As the focus currently lies on preparation and development of vaccines for the next Disease-X emergence, developing SoPs during preclinical and clinical development with appropriate animal models in conjunction with antibody transfer studies from human vaccine recipients will be key to fielding vaccines amidst a Disease-X outbreak and receiving the regulatory approval to utilize them.

## Future needs and CEPI funding

Having invested significantly in advancing CHIKV vaccines towards licensure, CEPI, with support from the European Commission, will continue to facilitate and drive access to licensed CHIKV vaccines in the future. By supporting Phase 4 effectiveness studies and further research to expand indications for licensed vaccines to pediatric, immunocompromised, and pregnant populations, CEPI will encourage developers to address gaps identified in this document as part of future grant proposals. This includes supporting technology transfers for manufacturing of CHIKV vaccines to endemic areas to ensure regional access and availability.

While these data become available over the coming years, we recognize CEPI’s limited mandate in market shaping and supply within the Global Health ecosystem. In this light we call on key partners such as WHO, PAHO, GAVI, UNICEF, National Regulatory Authorities, vaccine developers, and others to:

- Facilitate dialogue and visibility of advanced CHIKV vaccines at existing institutional fora and advisory bodies, such as SAGE, WHO PQ and others to facilitate uptake and use of these vaccines in advance of licensure.
- Highlight the need to streamline existing processes to facilitate access to vaccines through WHO PQ, GAVI, UNICEF and Revolving Fund procurement mechanisms, considering the significant operational burden on all key stakeholders.
- Encourage mechanisms such as joint regulatory review with affected country NRAs to ensure timely licensure of vaccines as an interim step to WHO PQ.
- Lay out and propose end-to-end interim mechanisms for regional emergency use of licensed (ML3-4 NRA) vaccines before completion of WHO PQ process.
- Propose use-principles for advanced and licensed vaccines by affected countries before a PQ pathway is available, such as, for outbreak and emergency use.
- Proactively engage with decision makers in affected countries to increase visibility and understanding of CHIKV vaccines and applications for use of licensed vaccines.
- Consider how learnings from CHIKV vaccine development can be applied to vaccine development for other WHO Priority pathogens such as MERS-CoV, Nipah, Lassa and Disease-X viruses.

### Supplementary information

Supplementary abbreviation list
