# Supplementary material for: Strategic considerations on developing a CHIKV vaccine and ensuring equitable access for countries in need
Source: NPJ Vaccines. 2023 Aug 18;8:123. doi: 10.1038/s41541-023-00722-x (PMC10439111; doi:10.1038/s41541-023-00722-x)
Supplement: Supplementary file 1 — Supplementary abbreviation list [file 41541_2023_722_MOESM1_ESM.pdf]

Perspective: Strategic considerations on developing a CHIKV vaccine

Abbreviation list

ANVISA	Agência Nacional de Vigilância Sanitária (Brazil)
AVAREF	Africa Vaccine Regulatory Forum
CEPI	Coalition for Epidemics Preparedness Innovations
CHIKV	chikungunya virus
DENV	Dengue virus
CBER	US Center for Biologics Evaluation and Research
DALYs	Disability-adjusted life years
DCGI	Drug Controller General of India
ELISA	Enzyme Linked Immunosorbent Assay
EC	European Commission
EID	Emerging Infectious Disease
EMA	European Medicines Agency
EPI	Expanded Programme on Immunization
FIND	Foundation for Innovative New Diagnostics
GAVI	Global Alliance for Vaccines and Immunization
GMTs	Geometric Mean Titers
MAYV	Mayaro virus
ML	Maturity Level
MSD	Merck, Sharp and Dohme
NHP	Non-Human Primate
NRA	National Regulatory Agency
ONNV	O'Nyong Nyong virus
FDA	US Food and Drug Administration
DoD	US Department of Defense
VRBPAC	Vaccines and Related Biological Products Advisory Committee

VLP	Virus-like Particle
PEI	Paul Ehrlich Institute
PAHO	Pan American Health Organization
RDT	Rapid Diagnostic Test
RT PCR	Real Time Polymerase Chain Reaction
SoP	Surrogate marker of protection
SRA	Stringent Regulatory Agency
UNICEF	United Nations Children's Fund
WHO	World Health Organization
EUL	WHO Emergency Use Listing
PQ	WHO Pre-qualification
SAGE	WHO Strategic Advisory Group of Experts on Immunization
WNV	West Nile virus
YFV	Yellow Fever virus
ZIKV	Zika virus
